# Diagnostic performance of hematological discrimination indices to discriminate between βeta thalassemia trait and iron deficiency anemia and using cluster analysis: Introducing two new indices tested in Iranian population

**DOI:** 10.1038/s41598-019-54575-3

**Published:** 2019-12-09

**Authors:** Mina Jahangiri, Fakher Rahim, Amal Saki Malehi

**Affiliations:** 1 Department of Biostatistics and Epidemiology, Faculty of Health, Ahvaz Jundishapur University of Medical Sciences, Ahvaz, Iran; 2 Ph.D. Student, Department of Biostatistics, Faculty of Medical Sciences, Tarbiat Modares University, Tehran, Iran; 3 Research Center of Thalassemia and Hemoglobinopathy, Health Research Institute, Ahvaz Jundishapur University of Medical Sciences, Ahvaz, Iran; 4 Clinical Research Development Unit, Golestan Hospital, Ahvaz Jundishapur University of Medical Sciences, Ahvaz, Iran

**Keywords:** Experimental models of disease, Experimental models of disease, Experimental models of disease, Outcomes research, Outcomes research

## Abstract

Although the discrimination between β-thalassemia trait (βTT) and Iron deficiency anemia (IDA) is important clinically, but it is challenging and normally difficult; so if a patient with IDA is diagnosed as βTT, then it is deprived of iron therapy. This study purpose was to evaluate the 26 different discriminating indices diagnostic function in patients with microcytic anemia by using accuracy measures, and also recommending two distinct new discriminating indices as well. In this study, 907 patients were enrolled with the ages over 18-year-old with either βTT or IDA. Twenty-six discrimination indices diagnostic performance presented in earlier studies, and two new indices were introduced in this study (CRUISE index and index26) in order to evaluate the differential between βTT and IDA by using accuracy measures. 537 (59%) patients with βTT (299 (56%) women, and 238 (44%) men), and also 370 (41%) patients with IDA (293 (79%) women, and 77 (21%) men) were participated in this study for evaluating the 28 discrimination indices diagnostic performance. Two new introduced indices (CRUISE index and index26) have better performance than some discrimination indices. Indices with the amount of AUC higher than 0.8 had very appropriate diagnostic accuracy in discrimination between βTT and IDA, and also CRUISE index has good diagnostic accuracy, too. The present study was also the first cluster analysis application in order to identify the homogeneous subgroups of different indices with similar diagnostic function. In addition, new indices that offered in this study have presented a relatively closed diagnostic performance by using cluster analysis for the different indices described in earlier studies. Thus, we suggest the using of cluster analysis in order to determine differential indices with similar diagnostic performances.

## Introduction

β-thalassemia trait (βTT) and iron deficiency anemia (IDA) are amongst the most regularly reported microcytic anemia disorders^1,2^. IDA is prevalent in developing countries, hence βTT is predominant in regions like the Mediterranean, the Middle East, and the South East^3–7^. However the discrimination between βTT and IDA is important clinically, but it is challenging and normally difficult, because both of the disorders are sometimes clinically and experimentally in the similar conditions^8–10^. Thus, if a patient with IDA is identified as βTT, then he is deprived of iron therapy. Considering that βTT does not need treatment, but the diagnosis of a patient with βTT, and IDA may cause attendant risk of birth of thalassemia major child in the pre-marriage genetic counseling^11–13^. To effectively differentiate between these two hematologic disorders, in addition to counting blood cells (CBC), also time-consuming, and cost-effective tests are essential. Because the definitive diagnosis between βTT and IDA is confirmed by performing blood tests in order to measure the HbA2, serum iron, serum ferritin, transferrin saturation, and total iron binding capacity (TIBC), and in fact these parameters are typically considered as the gold standards for discriminating between these two hematologic disorders^9,14–18^.

Because of the discriminating between these two disorders importance, and cost-effective and time-consuming tests in order to differentiate them, several discriminating indicators have been proposed in large-scale research for the rapid and inexpensive differentiation between these two common hematologic disorders since 1973. These indices are founded on the blood parameters obtained from automated cell counters of blood that traditionally derived parameters of Hb (Hemoglobin), Mean Corpuscular Volume (MCV), Mean Corpuscular Hemoglobin (MCH), Red Blood Cell Distribution Width (RDW), Mean Corpuscular Hemoglobin Concentration (MCHC), and Red Blood Cell Count (RBC)^19–41^. Several studies have studied these indices diagnostic accuracy, which presented different results, as well as none of these indicators showed a sensitivity and specificity of 100%^3,6,17,32,40,42–56.^ Therefore, this study purpose was to evaluate the diagnostic function of 26 different discriminating indices in patients with microcytic anemia, by using accuracy measures, and proposing two distinct new discriminating indices for differentiation between βTT and IDA, as well.

## Material and Methods

### Population evaluated to develop the new index

In this study, a total of 907 patients aged over 18 years old diagnosed with IDA or βTT were selected to develop new discriminating indices. Hematological parameters like Hb (Hemoglobin), Mean Corpuscular Volume (MCV), Mean Corpuscular Hemoglobin (MCH), Red Blood Cell Distribution Width (RDW), Mean Corpuscular Hemoglobin Concentration (MCHC), and Red Blood Cell count (RBC) were measured by using Sysmex kx-21 automated hematology analyzer.

### Inclusion criteria

In the IDA group, patients had hemoglobin (Hb) levels less than 12 and 13 g/dL for women and men, respectively. Mean corpuscular hemoglobin (MCH) and Mean corpuscular volume (MCV) were below 80 fL and 27 pg for both sexes, respectively, and for men, ferritin of <28 ng/mL was considered as IDA. In the βTT group, patients had a MCV value below 80 fL. Patients with HbA2 levels of >3.5% were considered as βTT carriers.

### Exclusion criteria

For the IDA group, patients who had mutations associated with αTT (3.7, 4.2, 20.5, MED, SEA, THAI, FIL, and Hph) were excluded so, individuals presenting the two diseases simultaneously were not selected. For the βTT group, patients with αTT confirmed by presence of mutations in molecular analysis were excluded. All patients with malignancies or inflammatory/infectious diseases diagnosed based on clinical data and personal information obtained from medical records were also excluded.

### Ethical consideration

This study was approved and supported by Ethical committee affiliated by the Ahvaz Jundishapur University of Medical Sciences (AJUMS), Ahvaz, Iran. A written informed consent was obtained before the enrollment. All methods were performed in accordance with the relevant guidelines and the institution regulations.

### Development of the new index

26 discrimination indices of diagnostic performance proposed in the literature, and 2 new indices introduced in this study (CRUISE index and index26) were considered for evaluation of differences between βTT and IDA using accuracy measures like sensitivity, specificity, false positive and negative rate, positive and negative predictive value, Youden’s index, accuracy, positive and negative likelihood ratio, diagnostic odds ratio (DOR) and area under the curve (AUC).

$${\rm{Sensitivity}}\,({\rm{True}}\,{\rm{Positive}}\,{\rm{Rate}})=\frac{{\rm{True}}\,{\rm{Positive}}}{({\rm{True}}\,{\rm{Positive}}\,+\,{\rm{False}}\,{\rm{Negative}})}$$

$${\rm{Specificity}}\,({\rm{True}}\,{\rm{Negative}}\,{\rm{Rate}})=\frac{{\rm{True}}\,{\rm{Negative}}}{({\rm{True}}\,{\rm{Negative}}\,+\,{\rm{False}}\,{\rm{Positive}})}$$

$${\rm{False}}\,{\rm{Negative}}\,{\rm{Rate}}=(1-{\rm{Sensitivity}})$$

$${\rm{False}}\,{\rm{Positive}}\,{\rm{Rate}}=(1-{\rm{Specificity}})$$

$${\rm{Positive}}\,{\rm{Predictive}}\,{\rm{Value}}\,({\rm{PPV}})=\frac{{\rm{True}}\,{\rm{Positive}}}{({\rm{True}}\,{\rm{Positive}}\,+\,{\rm{False}}\,{\rm{Positive}})}$$

$${\rm{Negative}}\,{\rm{Predictive}}\,{\rm{Value}}\,({\rm{NPV}})=\frac{{\rm{True}}\,{\rm{Negative}}}{({\rm{True}}\,{\rm{Negative}}\,+\,{\rm{False}}\,{\rm{Negative}})}$$

$${\rm{Youden}}\mbox{'}{\rm{s}}\,{\rm{Index}}={\rm{Sensitivity}}+{\rm{Specificity}}-1$$

$${\rm{Accuracy}}=\frac{({\rm{True}}\,{\rm{Negative}}+{\rm{True}}\,{\rm{Positive}})}{({\rm{True}}\,{\rm{Negative}}+{\rm{True}}\,{\rm{Positive}}+{\rm{False}}\,{\rm{Positive}}+{\rm{False}}\,{\rm{Negative}})}$$

$${\rm{PositiveLikelihood}}\,{\rm{Ratio}}\,({\rm{LR}}\,+\,)=\frac{{\rm{Sensitivity}}}{(1-{\rm{Specificity}})}$$

$${\rm{Negative}}\,{\rm{Likelihood}}\,{\rm{Ratio}}({\rm{LR}}\,-\,)=\frac{1-{\rm{Sensitivity}}}{{\rm{Specificity}}}$$

$${\rm{Diagnostic}}\,{\rm{Odds}}\,{\rm{Ratio}}({\rm{DOR}})=\frac{{\rm{Positive}}\,{\rm{Likelihood}}\,\mathrm{Ratio}\,}{{\rm{Negative}}\,{\rm{Likelihood}}\,\mathrm{Ratio}\,}$$

If a discrimination index had sensitivity, specificity, positive and negative predictive value, Youden’s index and accuracy near to 1, then this discrimination index has better differential performance. Discrimination index with likelihood ratio of greater than 10, negative likelihood ratio with lower than 0.1 and high diagnostic odds ratio has a good diagnostic performance in differentiation between βTT and IDA^57^. Also, receiver operating characteristic (ROC)^58^ curve analysis was used to calculate the AUC, and compare the amount of AUC of discrimination indices. AUC with higher value indicates an overall good performance measure for each discrimination index. A perfect diagnostic discrimination index has an AUC equal to 1. Relationship between the AUC with the diagnostic accuracy is defined as: 0.9 < AUC < 1: excellent, 0.8 < AUC < 0.9: very good, 0.7 < AUC < 0.8: good, 0.6 < AUC < 0.7: sufficient, 0.5 < AUC < 0.6: bad, AUC < 0.5: index not useful^57^.

Herein, 2 new discriminating indices (CRUISE index and index26) were proposed for differentiating between βTT and IDA. CRUISE index was created using CRUISE tree algorithm^59,60^, and important normalized variables were used for evaluating coefficients of hematological parameters in calculation of this index. Index26 was created by pooling all indices except the Janel (11 T) index. Index26 was computed similar to Janel (11 T) index^41^, but index26 was calculated by combination of 26 indices (all indices except Janel (11 T) index). Janel (11 T) index was calculated by combining some indices (England and Fraser, RBC, Mentzer, Shine and Lal, Srivastava, Green and King, RDW, RDWI, Ricerca, Ehsani, and Sirdah). Optimum cut off for index26 was calculated using Youden’s index (indeed, optimum cutoff has maximum Youden’s index).

Also cluster analysis was used in order to extract homogeneous groups of discrimination indices with a similar diagnostic performance, according to stated accuracy measures for determining the each discrimination index diagnostic performance.

Cluster analysis is a technique for extracting observations homogeneous subgroups in a data set containing n samples and P predictor variables. Different algorithms are recommended for cluster analysis and some of this algorithms are known as hierarchical algorithms like single-linkage, complete-linkage, average-linkage, Ward’s method, and k-means non-hierarchical algorithm^61^. In this study, we proposed the cluster analysis application by using accuracy measures as predictor variables and it can be an applicable idea for determining differential indices with a similar performances. In former studies, these indices were compared only in subjective way, according to the accuracy measures like sensitivity, specificity, positive and negative predictive value, positive and negative likelihood ratio, accuracy, Youden’s index and AUC^3,6,17,32,40,42,56^. We used hierarchical algorithm (complete-linkage), and also the optimal number of indices subgroups with a similar performances was selected by using the package of NbClust in R software. This package includes 30 appropriate measures for determining the subgroups optimal number. We selected the optimal number according to the majority role.

### Validation of the CRUISE Index and Index26

**Figure 1:**
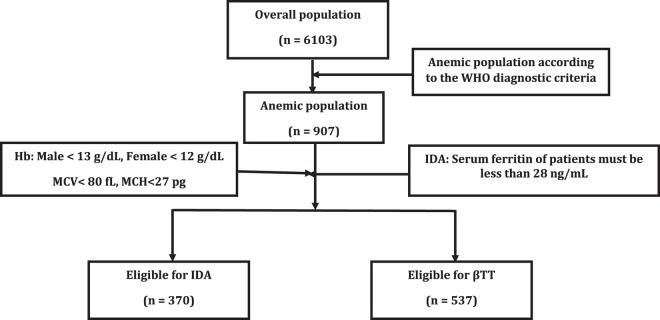
Design of study used for the validation of the CRUISE index and index26. Hb: hemoglobin; MCV: mean corpuscular volume; MCH: mean corpuscular hemoglobin; IDA: iron deficiency anemia; βTT: βeta thalassemia trait.

To validate the CRUISE index and index26, a cross-sectional study was performed in a referral center (Boghrat clinical center) in Tehran, Iran. A total of 6103 out-patients were screened among which 907 cases with anemia were included in this study. Classification of patients regarding having IDA or βTT was carried out according to the WHO diagnostic criteria^62^. Among 907 patients with anemia, 370 of them were eligible to have IDA and 537 of them were eligible to have βTT (Fig. 1).

### Statistical analysis

Descriptive statistics such as the mean, the standard deviation (SD), the median, and interquartile range (IQR) were calculated for hematological parameters and also age variable. Mann–Whitney U test was used in order to compare the differences between two groups parameters (βTT and IDA), because of these parameters distributions were non-normal. Normality of data was evaluated by using Shapiro-Wilk test. Sex variable was tested by chi-square test for both of the βTT and IDA groups.

Data were analyzed using a free statistical software named R version 5.3.0. Package epiR in R was used in order to calculate accuracy measures with their 95% exact confidence interval. ROC curve analysis was completed by using the package of pROC. Also, the package of OptimalCutpoints was used in order to calculate new discrimination indices cut off values by using Youden’s index. Determining the clusters optimal number, or homogeneous groups of diagnostic discrimination indices with similar performances was completed by using the package of NbClust. P < 0.05 was considered significant statistical difference.

## Result

537 (59%) patients with βTT (299 (56%) women and 238 (44%) men), and 370 (41%) patients with IDA (293 (79%) women, and 77 (21%) men) were participated in this research in order to evaluate the diagnostic performance of 28 discrimination indices (two of them are new indices like CRUISE index, and index26). Chi-square test pointed out that there is significant statistical association between sex and the disease groups (χ^2^(1) = 53.41, P < 0.001). Hematological parameters and age variable descriptive statistics of the study groups (βTT and IDA) are displayed in Table 1. According to information indicated in this table, we can concluded that all variables except HCT and RDW variables present significant difference amongst the groups (P < 0.001).

**Table 1:** Descriptive statistics of hematological parameters and age variable of study groups (IDA and βTT).

	βTT (n = 537)		IDA (n = 370)		P-value
	Mean ± SD	Median (IQR)	Mean ± SD	Median (IQR)	
Age	21.98 ± 16.37	20 (24)	28.86 ± 14.58	27 (22.75)	<0.001
MCV	62.17 ± 4.14	62 (5.4)	71.87 ± 6.93	72.2 (9.73)	<0.001
MCH	19.75 1.45	.196 (1.8)	21.85 ± 2.99	21.9 (4.2)	<0.001
MCHC	31.71 ± 1.48	31.84 (1.43)	30.40 ± 3.04	30.3 (2.71)	<0.001
Hb	11.20 ± 1.41	11 (1.16)	10.82 ± 2.43	10.45 (2.62)	<0.001
HCT	35.39 ± 4.73	34.6 (5.15)	35.53 ± 6.71	34 (7.65)	0.182
RDW	15.88 ± 1.43	15.7 (1.7)	16.04 ± 2.31	15.7 (3.32)	0.94
RBC	5.69 ± 0.67	5.61 (0.93)	4.91 ± 0.69	4.83 (0.83)	<0.001
HbA2	5.09 ± 0.74	5 (1.1)	2.43 ± 0.63	2.4 (0.83)	<0.001
Serum Iron	85.05 ± 32.96	86 (47)	25.66 ± 8.21	25 (13)	<0.001
TIBC	346.35 ± 47.02	345 (54)	480 ± 25.77	466 (40)	<0.001
Serum Ferritin	55.44 ± 56.64	38.9 (53.9)	4.52 ± 1.85	4.3 (2.3)	<0.001

Discrimination indices with their cut off are shown in Table 2. The number of true positive and negative, false positive and negative, and total number of correctly identified patients (true positive + true negative) are displayed in Table 3 for each discrimination index. Table 4 indicates sensitivity, specificity, false positive and negative rate, and positive and negative predictive values for 28 discrimination indices, and also in Table 5 the rank of these discrimination indices according to accuracy measures is shown.

**Table 2:** Discrimination indices for differential between βTT (n = 537) and IDA (n = 370) in patients with microcytic anemia.

Discriminant Formula	Reference	Calculation	Cut–off βTT	Cut–off IDA
England and Fraser (E&F)	^19^	MCV − RBC − (5 HB) − 3.4	<0	>0
RBC	^20^	RBC	>5	<5
Mentzer	^21^	MCV/RBC	<13	>13
Srivastava	^22^	MCH/RBC	<3.8	>3.8
Shine and Lal (S&L)	^23^	MCV × MCH × 0/01	<1530	>1530
Bessman	^24^	RDW	<14	>14
Ricerca	^25^	RDW/RBC	<4.4	>4.4
Green and King (G&K)	^26^	(MCV^2^ × RDW)/(100 HB)	<65	>65
Das Gupta	^27^	1.89 RBC − 0.33 RDW − 3.28	>0	<0
Jayabose (RDWI)	^28^	(MCV × RDW)/RBC	<220	>220
Telmissani – MCHD	^29^	MCH/MCV	<0.34	>0.34
Telmissani – MDHL	^29^	(MCH × RBC)/MCV	>1.75	<1.75
Huber– Herklotz	^30^	(MCH × RDW/10 RBC) + RDW	<20	>20
Kerman I	^31^	(MCV × MCH)/RBC	<300	300–400
Kerman II	^31^	(MCV × MCH × 10)/(RBC × MCHC)	<85	85–105
Sirdah	^32^	MCV − RBC − (3 Hb)	<27	>27
Ehsani	^33^	MCV − (10 RBC)	<15	>15
Keikhaei	^34^	(HB × RDW × 100)/(RBC^2^ × MCHC)	<21	>21
Nishad	^35^	0.615 MCV + 0.518 MCH + 0.446 RDW	<59	>59
Wongprachum	^36^	(MCV × RDW/RBC) – 10 HB	<104	>104
Sehgal	^37^	MCV^2^/RBC	<972	>972
Pornprasert	^38^	MCHC	<31	>31
Sirachainan	^39^	1.5 HB – 0.05 MCV	>14	<14
Bordbar	^40^	|80−MCV| × |27−MCH|	>44.76	<44.76
Matos and Carvalho (MC)	^64^	1.91 RBC + 0.44 MCHC	>23.85	<23.85
Janel (11 T)	^41^	Combination of RBC, Mentzer, S&L, E&F, Srivastava, G&K, RDW, RDWI, Ricerca, Ehsani and Sirdah	≥8	<8
CRUISE	MCHC + 0.603 RBC + 0.523 RDW		≥ 42.63	<42.63
Index26	Combination of all indices except Janel (11 T) index		≥ 16	<16

**Table 3:** True positive and negative (TP and TN), false positive and negative (FP and FN) and total number of correctly identified patients (TP + TN) of each discrimination index for differential between βTT (n = 537) and IDA (n = 370) in patients with microcytic anemia.

Discriminant Formula		TP	FP	FN	TN	(TP + TN)
England and Fraser (E&F)	βTM	338	54	199	316	654
	IDA	316	199	54	338	
RBC	βTM	464	137	73	223	687
	IDA	223	73	137	464	
Mentzer	βTM	478	79	59	291	769
	IDA	291	59	79	478	
Srivastava	βTM	402	71	135	299	701
	IDA	299	135	71	402	
Shine and Lal (S&L)	βTM	537	305	0	65	842
	IDA	65	0	305	537	
Bessman	βTM	34	74	503	296	330
	IDA	296	503	74	34	
Ricerca	βTM	530	344	7	26	556
	IDA	26	7	344	530	
Green and King (G&K)	βTM	465	79	72	291	756
	IDA	291	72	79	465	
Das Gupta	βTM	512	236	25	134	646
	IDA	134	25	236	512	
Jayabose (RDWI)	βTM	497	132	40	238	735
	IDA	238	40	132	497	
Telmissani – MCHD	βTM	528	357	9	13	541
	IDA	13	9	357	528	
Telmissani – MDHL	βTM	303	53	234	317	620
	IDA	317	234	53	303	
Huber – Herklotz	βTM	121	52	416	318	439
	IDA	318	416	52	121	
Kerman I	βTM	507	141	30	229	736
	IDA	229	30	141	507	
Kerman II	βTM	476	66	61	304	780
	IDA	304	61	66	476	
Sirdah	βTM	431	42	106	328	759
	IDA	328	106	42	431	
Ehsani	βTM	478	69	59	301	779
	IDA	301	59	69	478	
Keikhaei	βTM	476	101	61	269	745
	IDA	269	61	101	476	
Nishad	βTM	458	85	79	285	743
	IDA	285	79	85	458	
Wongprachum	βTM	472	113	65	257	729
	IDA	257	65	113	472	
Sehgal	βTM	516	131	21	239	755
	IDA	239	21	131	516	
Pornprasert	βTM	110	237	427	133	243
	IDA	133	427	237	110	
Sirachainan	βTM	193	93	344	277	470
	IDA	277	344	93	193	
Bordbar	βTM	522	165	15	205	727
	IDA	205	15	165	522	
Matos and Carvalho(MC)	βTM	422	76	115	294	716
	IDA	294	115	76	422	
Janel (11T)	βTM	423	38	114	332	755
	IDA	332	114	38	423	
CRUISE	βTM	413	102	124	268	682
	IDA	268	124	102	413	
Index26	βTM	424	26	113	344	766
	IDA	344	113	26	424

**Table 4:** Sensitivity (TPR), specificity (TNR), false positive and negative rate (FNR and FPR), positive and negative predictive values (PPV and NPV) of each discrimination index for differential βTT (n = 537) from IDA (n = 370) in patients with microcytic anemia with their 95% exact confidence interval.

Discriminant Formula	TPR (%)	TNR (%)	FNR (%)	FPR (%)	PPV (%)	NPV (%)
England and Fraser (E&F)	62.94 (58.70–67.04)	85.41 (81.39–88.84)	37.06 (32.96–41.30)	14.59 (11.16–18.61)	86.22 (82.41–89.48)	61.36 (57–65.59)
RBC	86.41 (83.21–89.19)	61.94 (56.71–66.98)	13.59 (10.81–16.79)	38.06 (33.02–43.29)	77.20 (73.64–80.50)	75.34 (70.02–80.14)
Mentzer	89.01 (86.06–91.53)	78.65 (74.12–82.72)	10.99 (8.47–13.94)	21.35 (17.28–25.88)	85.82 (82.64–88.61)	83.14 (78.80–86.91)
Srivastava	74.86 (70.97–78.48)	80.81 (76.42–84.70)	25.14 (21.52–29.03)	19.19 (15.30–23.58)	84.99 (81.45–88.09)	68.89 (64.31–73.22)
Shine and Lal (S&L)	100 (99.32–100)	17.57 (13.83–21.84)	0 (0–0.68)	82.43 (78.16–86.17)	63.78 (60.43–67.03)	100 (94.48–100)
Bessman	6.33 (4.42–8.72)	80 (75.56–83.96)	93.67 (91.28–95.58)	20 (16.04–24.44)	31.48 (22.88–41.13)	37.05 (33.69–40.50)
Ricerca	98.70 (97.33–99.47)	7.03 (4.64–10.13)	1.30 (0.53–2.67)	92.97 (89.87–95.36)	60.64 (57.31–63.90)	78.79 (61.09–91.02)
Green and King (G&K)	86.59 (83.42–89.36)	78.65 (74.12–82.72)	13.41 (10.64–16.58)	21.35 (17.28–25.88)	85.48 (82.23–88.33)	80.17 (75.69–84.14)
Das Gupta	95.34 (93.20–96.96)	36.22 (31.31–41.34)	4.66 (3.04–6.8)	63.78 (58.66–68.69)	68.45 (64.98–71.77)	84.28 (77.67–89.56)
Jayabose (RDWI)	92.55 (89.99–94.63)	64.32 (59.21–69.21)	7.45 (5.37–10.01)	35.68 (30.79–40.79)	79.01 (75.62–82.13)	85.61 (80.93–89.52)
Telmissani–MCHD	98.32 (96.84–99.23)	3.51 (1.88–5.93)	1.68 (0.77–3.16)	96.49 (94.07–98.12)	59.66 (56.34–62.91)	59.09 (36.35–79.29)
Telmissani–MDHL	56.42 (52.11–60.67)	85.68 (81.69–89.08)	43.58 (39.33–47.89)	14.32 (10.92–18.31)	85.11 (80.98–88.65)	57.53 (53.28–61.70)
Huber– Herklotz	22.53 (19.07–26.31)	85.95 (81.98–89.32)	77.47 (73.69–80.93)	14.05 (10.68–18.02)	69.94 (62.52–76.67)	43.32 (39.70–47)
Kerman I	94.41 (92.12–96.20)	61.89 (56.73–66.86)	5.59 (3.8–7.88)	38.11 (33.14–43.27)	78.24 (74.86–81.36)	88.42 (83.88–92.05)
Kerman II	88.64 (85.65–91.20)	82.16 (77.87–85.93)	11.36 (8.80–14.35)	17.84 (14.07–22.13)	87.82 (84.77–90.46)	83.29 (79.06–86.97)
Sirdah	80.26 (76.64–83.55)	88.65 (84.97–91.70)	19.74 (16.45–23.36)	11.35 (8.30–15.03)	91.12 (88.19–93.53)	75.58 (71.25–79.55)
Ehsani	89.01 (86.06–91.53)	81.35 (77–85.19)	10.99 (8.47–13.94)	18.65 (14.81–23)	87.39 (84.31–90.05)	83.61 (79.37–87.28)
Keikhaei	88.64 (85.65–91.20)	72.70 (67.86–77.18)	11.36 (8.8–14.35)	27.30 (22.82–32.14)	82.50 (79.14–85.51)	81.52 (76.90–85.56)
Nishad	85.29 (82.01–88.18)	77.03 (72.40–81.22)	14.71 (11.82–17.99)	22.97 (18.78–27.60)	84.35 (81.01–87.30)	78.30 (73.70–82.42)
Wongprachum	87.90 (84.83–90.53)	69.46 (64.49–74.12)	12.10 (9.47–15.17)	30.54 (25.88–35.51)	80.68 (77.25–83.81)	79.81 (75.01–84.06)
Sehgal	96.09 (94.08–97.56)	64.59 (59.48–69.47)	3.91 (2.44–5.92)	35.41 (30.53–40.52)	79.75 (76.45–82.78)	91.92 (87.92–94.93)
Pornprasert	20.48 (17.15–24.15)	35.95 (31.05–41.07)	79.52 (75.85–82.85)	64.05 (58.93–68.95)	31.70 (26.84–36.88)	23.75 (20.28–27.50)
Sirachainan	35.94 (31.88–40.16)	74.86 (70.12–79.21)	64.06 (59.84–68.12)	25.14 (20.79–29.88)	67.48 (61.72–72.88)	44.61 (40.65–48.61)
Bordbar	97.21 (95.43–98.43)	55.40 (50.18–60.54)	2.79 (1.54–4.57)	44.59 (39.46–49.82)	75.98 (72.61–79.13)	93.18 (89–96.13)
Matos and Carvalho	78.58 (74.87–81.98)	79.46 (74.98–83.46)	21.42 (18.02–25.13)	20.54 (16.54–25.02)	84.74 (81.27–87.78)	71.88 (67.26–76.19)
Janel (11 T)	78.77 (75.07–82.16)	89.73 (86.18–92.63)	21.23 (17.84–24.93)	10.27 (7.37–13.82)	91.76 (88.86–94.10)	74.44 (70.13–78.43)
CRUISE	76.91 (73.11–80.41)	72.43 (67.58–76.93)	23.09 (19.59–26.89)	27.57 (23.07–32.42)	80.19 (76.49–83.55)	68.37 (63.51–72.95)
Index26	78.96 (75.26–82.33)	92.97 (89.87–95.36)	21.04 (17.67–24.74)	7.03 (4.64–10.13)	94.22 (91.65–96.19)	75.27 (71.05–79.16)

**Table 5:** Ranking of diagnostic performance of discrimination indices for differential βTT (n = 537) from IDA (n = 370) in patients with microcytic anemia based on sensitivity (TPR), specificity (TNR), positive and negative predictive values (PPV and NPV), Youden’s index, accuracy, diagnostic odds ratio (DOR) and area under the curve (AUC) (lower rank shows better diagnostic performance).

Discriminant Formula	TPR	TNR	PPV	NPV	Youden’s Index	Accuracy	DOR	AUC
England and Fraser (E&F)	23	6	6	22	19	19	19	19
RBC	15	21	19	16	19	17	18	18
Mentzer	9.5	13	7	9	6	3	8	6
Srivastava	22	9	10	20	15	16	16	15
Shine and Lal (S&L)	1	26	24	1	22	22		22
Bessman	28	10	28	27	27	27	26	27
Ricerca	2	27	25	13	25	23	22	25
Green and King (G&K)	14	13	8	11	7	6	10	7
Das Gupta	6	24	22	6	21	20	17	21
Jayabose (RDWI)	8	20	17	5	13	12	11	13
Telmissani – MCHD	3	28	26	23	26	24	23	26
Telmissani – MDHL	24	5	9	24	20	21	21	20
Huber – Herklotz	26	4	21	26	24	26	24	24
Kerman I	7	22	18	4	14	11	9	14
Kerman II	11.5	7	4	8	2	1	4	2
Sirdah	17	3	3	15	4	5	6	4
Ehsani	9.5	8	5	7	3	2	5	3
Keikhaei	11.5	16	13	10	9	9	12	9
Nishad	16	14	12	14	8	10	13	8
Wongprachum	13	18	14	12	12	13	14	12
Sehgal	5	19	16	3	10	8	2	10
Pornprasert	27	25	27	28	28	28	27	28
Sirachainan	25	15	23	25	23	25	25	23
Bordba	4	23	20	2	16	14	3	16
Matos and Carvalho	20	11	11	19	11	15	15	11
Janel (11 T)	19	2	2	18	5	8	7	5
CRUISE	21	17	15	21	17	18	20	18
Index26	18	1	1	17	1	4	1	1

Table 4 represents that none of discrimination indices have 100% specificity and 100% positive predictive value. Also, none of indices except Shine and Lal (S&L) have 100% sensitivity and 100% negative predictive value, but this index has very high false positive rate. According to information indicated in the Table 4 and the Table 5, Shine and Lal (S&L) and Bessman point out the highest and lowest sensitivity (the lowest and highest false negative rate) in βTT diagnose, respectively, and index26 and Telmissani–MCHD index indicate the highest and lowest specificity (the lowest and highest false positive rate) in IDA diagnose, respectively. Also index26 and Bessman showed the highest and lowest positive predictive value, respectively, and Shine and Lal (S&L) and Pornprasert had highest and lowest negative predictive value (Table 4 and Table 5).

Table 5 and Table 6 presented that lowest Youden’s index is related to the Pornprasert, and the highest amount is related to the index26. Also, these tables show that KermanII and Pornprasert have the highest and lowest accuracy, respectively, and the highest DOR is belong to index26, and the lowest is belong to Pornprasert. Two new indices introduced earlier (CRUISE index and index26), have better performance than some of the discrimination indices, which were listed in Table 2 (Table 5). Due to the findings, none of indices have LR + > 10, and only KermanI index has LR − <0.1.

**Table 6:** Youden’s index, accuracy, positive and negative likelihood ratio (LR+ and LR−) and diagnostic odds ratio (DOR) of each discrimination index for differential βTT (n = 537) from IDA (n = 370) in patients with microcytic anemia with their 95% exact confidence interval.

Discriminant Formula	Youden’s Index (%)	Accuracy (%)	LR + (%)	LR − (%)	DOR (%)
England and Fraser (E&F)	48.35 (40.09–55.88)	72.11 (69.06–75)	4.31 (3.34–5.56)	0.43 (0.39–0.49)	10.02 (7.092–13.93)
RBC	48.35 (39.92–56.17)	76.59 (73.68–79.32)	2.27 (1.98–2.60)	0.22 (0.17–0.28)	10.32 (7.47–14.33)
Mentzer	67.66 (60.17–74.25)	84.78 (82.28–87.06)	4.17 (3.42–5.08)	0.14 (0.11–0.18)	29.79 (20.67–43.09)
Srivastava	55.67 (47.39–63.17)	77.29 (74.42–79.98)	3.90 (3.15–4.84)	0.31 (0.27–0.36)	12.58 (9.07–17.34)
Shine and Lal (S&L)	17.57 (12.80–21.83)	66.37 (63.19–69.44)	1.21 (1.16–1.27)	0	∞
Bessman	–13.67 (–20.02–7.31)	36.38 (33.25–39.61)	0.32 (0.22–0.46)	1.17 (1.11–1.24)	0.27 (0.18–0.42)
Ricerca	5.72 (1.97–9.60)	61.30 (58.04–64.48)	1.06 (1.03–1.09)	0.19 (0.08–0.42)	5.58 (2.46–13.33)
Green and King (G&K)	65.24 (57.53–72.08)	83.35 (80.76–85.72)	4.06 (3.33–4.95)	0.17 (0.14–0.21)	23.88 (16.74–33.80)
Das Gupta	31.56 (24.52–38.31)	71.22 (68.16–74.15)	1.49 (1.38–1.62)	0.13 (0.09–0.19)	11.46 (7.38–18.31)
Jayabose (RDWI)	56.87 (49.20–63.83)	81.04 (78.33–83.54)	2.59 (2.26–2.98)	0.12 (0.09–0.16)	21.58 (15.23–32.96)
Telmissani – MCHD	1.83 (–1.27–5.16)	59.65 (56.37–62.86)	1.02 (1.00–1.04)	0.48 (0.21–1.10)	2.13 (0.90–5.05)
Telmissani – MDHL	42.10 (33.80–49.75)	68.36 (65.22–71.37)	3.94 (3.04–5.11)	0.51 (0.46–0.56)	7.73 (5.53–10.85)
Huber – Herklotz	8.48 (1.05–15.63)	48.40 (45.10–51.71)	1.60 (1.19–2.16)	0.90 (0.85–0.96)	1.78 (1.25–2.54)
Kerman I	56.30 (48.85–63.06)	81.15 (78.45–83.64)	2.48 (2.17–2.83)	0.09 (0.06–0.13)	27.56 (17.97–41.94)
Kerman II	70.80 (63.52–77.13)	86.00 (83.57–88.19)	4.97 (3.98–6.20)	0.14 (0.11–0.18)	35.50 (24.66–52.38)
Sirdah	68.91 (61.61–75.24)	83.68 (81.11–86.03)	7.07 (5.30–9.43)	0.22 (0.19–0.27)	32.14 (21.60–46.67)
Ehsani	70.36 (63.06–76.72)	85.89 (83.45–88.09)	4.77 (3.85–5.92)	0.14 (0.11–0.17)	34.07 (24.26–51.49)
Keikhaei	61.34 (53.51–68.38)	82.14 (79.49–84.58)	3.25 (2.74–3.85)	0.16 (0.12–0.20)	20.31 (14.63–29.53)
Nishad	62.32 (54.40–69.39)	81.92 (79.26–84.37)	3.71 (3.07–4.49)	0.19 (0.15–0.24)	19.53 (13.83–27.31)
Wongprachum	57.36 (49.32–64.65)	80.38 (77.64–82.91)	2.88 (2.46–3.37)	0.17 (0.14–0.22)	16.94 (11.75–23.22)
Sehgal	60.68 (53.57–67.03)	83.24 (80.65–85.62)	2.71 (2.36–3.12)	0.06 (0.04–0.09)	45.17 (27.59–72.85)
Pornprasert	–43.57 (–51.80 – –34.78)	26.79 (23.93–29.80)	0.32 (0.27–0.38)	2.21 (1.92–2.55)	0.15 (0.11–0.20)
Sirachainan	10.80 (2–19.37)	51.82 (48.51–55.12)	1.43 (1.16–1.76)	0.86 (0.78–0.93)	1.66 (1.25–2.24)
Bordbar	52.61 (45.61–58.97)	80.15 (77.41–82.70)	2.18 (1.94–2.44)	0.05 (0.03–0.08)	43.60 (24.88–75.14)
Matos and Carvalho	58.04 (49.85–65.44)	78.94 (76.14–81.55)	3.83 (3.12–4.70)	0.27 (0.23–0.32)	14.20 (10.25–19.66)
Janel (11T)	68.50 (61.24–74.79)	83.24 (80.65–85.62)	7.67 (5.66–10.40)	0.24 (0.20–0.28)	31.96 (21.86–48.09)
CRUISE	49.34 (40.69–57.33)	75.08 (72.13–77.87)	2.79 (2.35–3.31)	0.32 (0.27–0.38)	8.72 (6.46–11.86)
Index26	71.93 (65.13–77.69)	84.67 (82.16–86.96)	11.24 (7.74–16.32)	0.23 (0.19–0.27)	48.87 (31.67–77.81)

**Figure 2:**
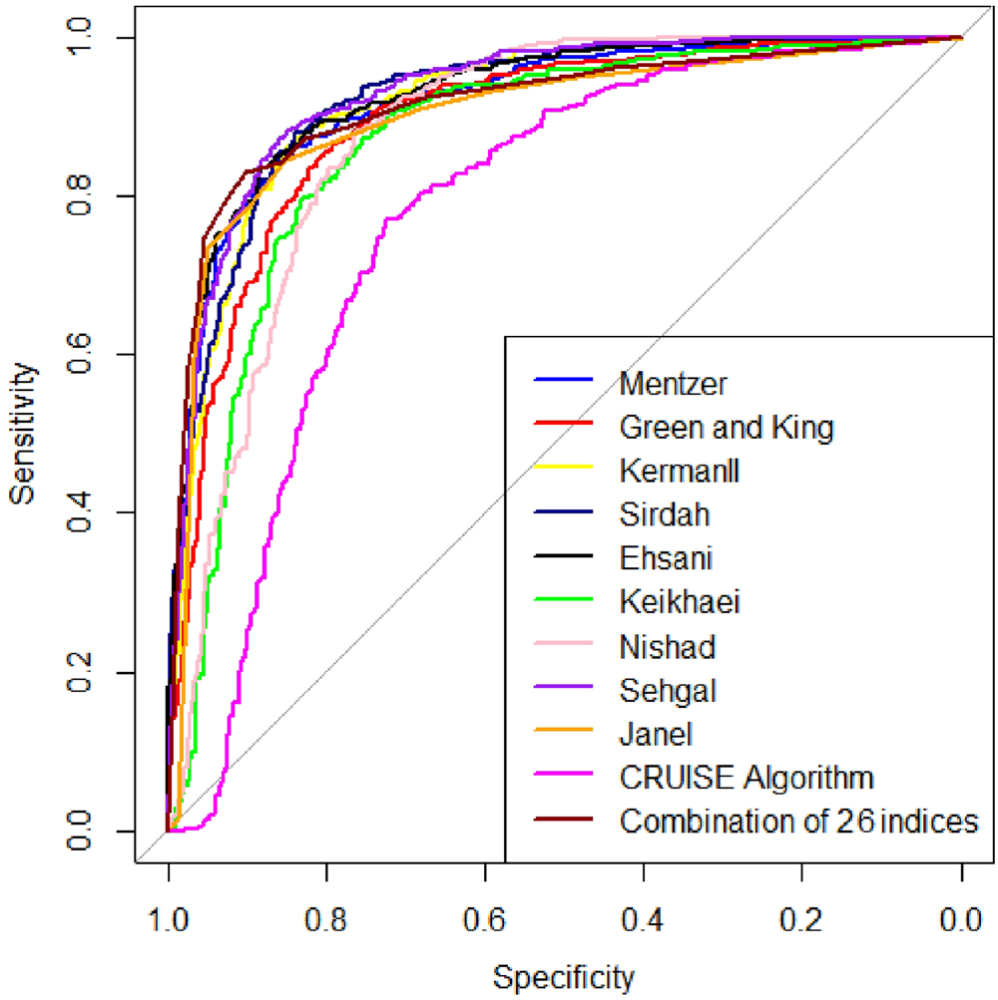
Reciever operating characteristic curves of discrimination indices with area under curve (AUC) higher than 0.8 (discrimination indices such as: index26, Kerman II, Ehsani, Sirdah, Janel (11T), Mentzer, Green and King (G&K), Nishad, Keikhaei, Sehgal and CRUISE).

Each discrimination index AUC is shown in Table 7. Also, Fig. 2 showed the ROC curves for discrimination formula with the amount of AUC higher than 0.8 (Kerman II, Ehsani, Sirdah, Janel (11 T), Mentzer, Green and King (G&K), Nishad, Keikhaei and Sehgal), and two new indices (CRUISE index and index26). Indices with the amount of AUC higher than 0.8 have very appropriate diagnostic accuracy in the discrimination between βTT and IDA, and also CRUISE index has good diagnostic accuracy. AUC of all indices except Telmissani–MCHD were statistically significant, in regard to the amount of AUC equal to 0.5 (P < 0.001) (Table 7), and AUC of Bessman and Pornprasert were significantly less than 0.5 (P < 0.001). As shown in Tables 5 and 7, the highest AUC is related to index26, and the lowest AUC is related to the Pornprasert index. Comparison between AUCs of discrimination formula (indices with AUC higher than 0.8), and two new indices are displayed in Table 8. There was a significant difference between AUC of CRUISE index and other indices, which the AUC of this index was significantly less than other indices (P < 0.001) (Table 8), but this index has higher AUC than the amount of other indices recorded in Table 2 (Table 7). Table 8 also represented that the AUC of index26 is significantly higher than Green and King (G&K), Keikhaei, Nishad, Sehgal, Janel (11 T) and CRUISE index (P < 0.05), but there is no significant difference between AUC of this index and other indices like Mentzer, Kerman II, Ehsani and Sirdah (P > 0.05).

**Table 7:** Area under the curve (AUC) of each discrimination index for differential βTT (n = 537) from IDA (n = 370) in patients with microcytic anemia with their 95% confidence interval (SE: Standard Error, CI: Confidence Interval).

Discriminant Formula	AUC	SE	95% CI	p–value
England and Fraser (E&F)	0.742	0.0139	0.714–0.769	<0.001
RBC	0.747	0.0146	0.718–0.775	<0.001
Mentzer	0.838	0.0126	0.814–0.863	<0.001
Srivastava	0.778	0.0139	0.751–0.806	<0.001
Shine and Lal (S&L)	0.588	0.0099	0.568–0.607	<0.001
Bessman	0.432	0.0117	0.409–0.455	<0.001
Ricerca	0.529	0.0071	0.515–0.542	<0.001
Green and King (G&K)	0.826	0.0130	0.801–0.852	<0.001
Das Gupta	0.658	0.0133	0.632–0.684	<0.001
Jayabose (RDWI)	0.784	0.0137	0.757–0.811	<0.001
Telmissani – MCHD	0.509	0.0055	0.498–0.520	0.0970
Telmissani – MDHL	0.711	0.0141	0.683–0.738	<0.001
Huber – Herklotz	0.542	0.0128	0.517–0.567	0.001
Kerman I	0.782	0.0136	0.755–0.808	<0.001
Kerman II	0.854	0.0121	0.830–0.878	<0.001
Sirdah	0.845	0.0119	0.821–0.868	<0.001
Ehsani	0.852	0.0122	0.828–0.876	<0.001
Keikhaei	0.807	0.0135	0.780–0.833	<0.001
Nishad	0.812	0.0134	0.785–0.838	<0.001
Wongprachum	0.787	0.0139	0.759–0.814	<0.001
Sehgal	0.803	0.0131	0.778–0.829	<0.001
Pornprasert	0.282	0.018	0.247–0.317	<0.001
Sirachainan	0.554	0.0153	0.524–0.584	0.0004
Bordbar	0.763	0.0134	0.737–0.789	<0.001
Matos and Carvalho	0.790	0.0138	0.763–0.817	<0.001
Janel (11T)	0.843	0.0119	0.819–0.866	<0.001
CRUISE	0.747	0.0148	0.718–0.776	<0.001
Index26	0.858	0.0111	0.836–0.879	<0.001

**Table 8:** Comparison between area under the curve (AUC) values of discrimination indices with AUC higher than 0.8 for differential βTT (n = 537) from IDA (n = 370) in patients with microcytic anemia (AUC_d_ = AUC_row_ – AUC_column_, SE: Standard Error (AUC_d_)).

	G&K	Mentzer	Kerman II	Sirdah	Ehsani	Keikhaei	Nishad	Sehgal	Janel (11 T)	CRUISE
Mentzer	AUC_d_ = 0.012 SE = 0.0145 P = 0.404									
Kerman II	AUC_d_ = 0.028 SE = 0.0156 P = 0.074	AUC_d_ = 0.016 SE = 0.009 P = 0.0810								
Sirdah	AUC_d_ = 0.018 SE = 0.0125 P = 0.142	AUC_d_ = 0.006 SE = 0.0111 P = 0.575	AUC_d_ = –0.009 SE = 0.0125 P = 0.450							
Ehsani	AUC_d_ = 0.026 SE = 0.015 P = 0.089	AUC_d_ = 0.013 SE = 0.0057 P = 0.017	AUC_d_ = –0.002 SE = 0.0073 P = 0.763	AUC_d_ = 0.007 SE = 0.0114 P = 0.524						
Keikhaei	AUC_d_ = –0.019 SE = 0.0094 P = 0.039	AUC_d = _–0.0316 SE = 0.0136 P = 0.02	AUC_d_ = –0.047 SE = 0.0146 P = 0.001	AUC_d_ = –0.038 SE = 0.0134 P = 0.005	AUC_d_ = –0.045 SE = 0.0142 P = 0.001					
Nishad	AUC_d_ = –0.015 SE = 0.0183 P = 0.425	AUC_d_ = –0.027 SE = 0.0141 P = 0.057	AUC_d_ = –0.042 SE = 0.0119 P = 0.0004	AUC_d_ = –0.033 SE = 0.0161 P = 0.0411	AUC_d_ = –0.040 SE = 0.0131 P = 0.002	AUC_d_ = 0.005 SE = 0.0181 P = 0.788				
Sehgal	AUC_d_ = –0.023 SE = 0.017 P = 0.18	AUC_d_ = –0.035 SE = 0.0116 P = 0.003	AUC_d_ = –0.051 SE = 0.012 P < 0.001	AUC_d_ = –0.041 SE = 0.0149 P = 0.006	AUC_d_ = –0.048 SE = 0.0112 P < 0.001	AUC_d_ = –0.003 SE = 0.0165 P = 0.841	AUC_d_ = –0.008 SE = 0.0124 P = 0.51			
Janel (11 T)	AUC_d_ = 0.0163 SE = 0.012 P = 0.176	AUC_d_ = 0.004 SE = 0.0111 P = 0.707	AUC_d_ = –0.011 SE = 0.0124 P = 0.355	AUC_d_ = –0.002 SE = 0.0061 P = 0.738	AUC_d = _–0.009 SE = 0.0115 P = 0.416	AUC_d_ = 0.036 SE = 0.0123 P = 0.004	AUC_d = _0.031 SE = 0.0162 P = 0.057	AUC_d_ = 0.039 SE = 0.0148 P = 0.008		
CRUISE	AUC_d_ = –0.08 SE = 0.0166 P < 0.001	AUC_d_ = –0.092 SE = 0.0184 P < 0.001	AUC_d_ = –0.107 SE = 0.0186 P < 0.001	AUC_d_ = –0.098 SE = 0.0167 P < 0.001	AUC_d_ = –0.105 SE = 0.0185 P < 0.001	AUC_d_ = –0.06 SE = 0.0178 P = 0.0008	AUC_d_ = –0.065 SE = 0.0209 P = 0.0019	AUC_d_ = –0.057 SE = 0.0191 P = 0.0029	AUC_d_ = –0.096 SE = 0.0172 P < 0.001	
Index26	AUC_d_ = 0.033 SE = 0.0125 P = 0.0076	AUC_d_ = 0.021 SE = 0.0112 P = 0.0566	AUC_d_ = 0.006 SE = 0.0115 P = 0.6231	AUC_d_ = 0.015 SE = 0.008 P = 0.0627	AUC_d_ = 0.008 SE = 0.0107 P = 0.4625	AUC_d_ = 0.053 SE = 0.0124 P < 0.001	AUC_d_ = 0.048 SE = 0.0153 P = 0.0017	AUC_d_ = 0.056 SE = 0.0143 P = 0.0001	AUC_d_ = 0.017 SE = 0.006 P = 0.0044	AUC_d_ = 0.113 SE = 0.0177 P < 0.001

**Figure 3:**
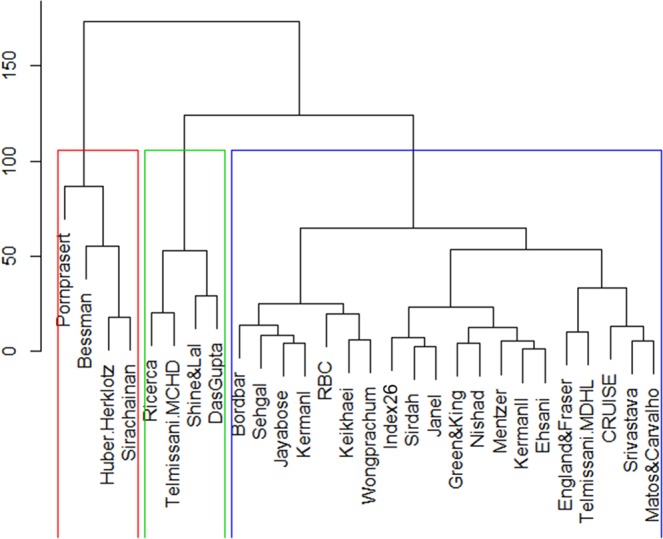
Dendrogram from cluster analysis for extracting homogeneous groups of diagnostic discrimination indices with similar performance (each rectangles includes diagnostic discrimination indices with similar performance).

Cluster analysis dendrogram (this plot represents steps in the cluster analysis) is presented in Fig. 3. Cluster analysis extracted three homogenous groups. First one of them includes discrimination indices like Pornprasert, Bessman, Huber –Herklotz, and Sirachainan. Second group includes Ricerca, Telmissani–MCHD, Shine and Lal (S&L), Das Gupta, and the third group includes discrimination indices like Bordbar, Sehgal, Jayabose, KermanI, RBC, Keikhaei, Wongprachum, Index26, Sirdah, Janel (11 T), Green and King (G&K), Nishad, Mentzer, KermanII, Ehsani, England and Fraser (E&F), Telmissani–MDHL, Srivastava, CRUISE. So two new introduced indices in this study have similar performances to indices of third homogenous group.

## Discussion

βTT and IDA are known as common causes for microcytic anemia, and these two hematologic disorders typically have similar clinical and experimental conditions. The definitive diagnostic method for the βTT is based on the HbA2 increase^17,18^, and the principal methods for diagnosis of IDA based on the increase in TIBC, as same as a decrease in serum iron, serum ferritin, and transferrin saturation^9^.

The exact discrimination between these two hematologic disorders is very vital, because the correct treatment and its proper diagnosis through premarital genetic counseling, would prevent the attendant risk of thalassemia major child birth. Considering the importance of differentiating between βTT and IDA, several different indices have been proposed in large-scale researches; additionally, these indices showed different diagnostic performance, and none of these indices had definitive diagnosis in various studies.

It is possible to discriminate between βTT and IDA without using expensive tests with high performance index. We presented two new discriminating indices between these two common microcytic anemia, and also compared these two indicators performance with 26 different published indices. This study findings indicated that none of the discriminating indices provided 100% sensitivity and specificity. Consequently, the Shine and Lal index showed a sensitivity and a negative predictive value, but with respect to the AUC, it had a poor performance in the differentiation between the βTT and IDA. It is important to remember that this index has expressed as the best discriminating index for differentiation between βTT and IDA in former researches^[9,50,63^. Shen *et al*., reported that S & L index had a low AUC as same as this study^55^. In the present study, index26 had 100% specificity and complete positive predictive value. In addition, according to Youden’s index, DOR, and AUC, this index is a differential index with superior performance for differentiation between the βTT and IDA. Accuracy measure like Youden’s index, accuracy, DOR, and AUC take both sensitivity and specificity into consideration, so they can present the discrimination indices performance more accurately than other criteria. According to these criteria and also Table 6, index26 indicates better performance in comparison to the other discrimination indices.

Also, by comparing the AUCs of various discriminating indices, this test performance was better than the differential indices significantly, like Green and King, Keikhaei, Nishad, Sehgal and Janel (11 T). Considering the worth of index26 in this study, this index is still difficult to calculate, and we are developing a calculator-based approach on differential indices expressed in the results, and in the future works we will introduce this protocol, in order to solve this problem. By using this calculator, we can determine the accuracy and each indicator outcome easily and quickly. Thus, it can be concluded that the differential indices, including Mentzer, Kerman II, Ehsani, Sirdah, janel (11 T) and index26 are reliable indices for discrimination between the βTT and IDA. Another recommended index was CRUISE, which showed a good diagnostic performance, but its AUC was significantly lower compared to the other indices with the very appropriate diagnostic performance (AUC > 0.8). As a result, this index has a superior performance compared to some of before stated indices. Several studies proposed new discrimination indices by using discriminant analysis for differentiating between the βTT and IDA (these indices are Nishad, Matos and Carvalho, Sirachainan and Das Gupta)^27,35,39,64,65^. We used CRUISE tree algorithm for recommending a new discrimination index, because tree-based methods are non-parametric methods, and these methods have some advantages over the traditional statistical methods like discriminant analysis. Some of these advantages are known as following: without needing to determine assumptions about the functional form between outcome variable and predictor variables, useful for dealing with nonlinear relationships and high-order interactions, and robust to outliers and multicollinearity. In this study, CRUISE index showed a high AUC in comparison with the Sirachainan and Das Gupta indices.

Different studies are conducted in order to assess the differential indices diagnostic performance for discriminating between the βTT and IDA in different populations. Also, these studies indicated different results. We mention index with best diagnostic performance based on the highest AUC or Youden’s index here in some conducted studies in different populations.

Iranian population: Ghafouri *et al*. in 2006^46^: Mentzer index, Rahim and Keikhaei in 2009^45^: Shine and Lal index in patients < 10 years and RDW and RDWI index in patients with the age of 10 to 57 years old, Ehsani *et al*. in 2009^33^: Mentzer index and Ehsani index, Ahmadi *et al*. in 2009^44^: Shine and Lal index, Keikhaei in 2010^34^: Keikhaei index, Sargolzaie and Miri-Moghaddam in 2014^53^: Green and King index, Bordbar *et al*. in 2015^40^: Bordbar index. Thailand population: Sirachainan *et al*. in 2014^39^: Sirachainan index. Indian population: Tripathi *et al*. in 2015^66^: Mentzer index, Piplani *et al*. in 2016^67^: Mentzer index. Turkey population: Demir *et al*. in 2002^17^: RBC index, Beyan *et al*. in 2007^48^: RBC index, Vehapoglu *et al*. 2014^56^: Mentzer index. Italy population: Ferrara *et al*. in 2010^68^: England and Fraser index. Kuwait population: AlFadhli *et al*. in 2006^49^: England and Fraser index. Sri Lanka population: Nishad *et al*. in 2012^35^: Nishad index. Palestinian population: Sirdah *et al*. in 2007^32^: Sirdah index. Brazilian population: Matos *et al*. in 2013^54^: Green and King index. Chinese population: Shen *et al*. in 2010^55^: Green and King index. France population: Janel *et al*. in 2011^41^: 11 T, Green and King, RDWI and Sirdah index. Saudi Arabia population: Jameel *et al*. in 2017^69^: RDWI index.

### Conclusion and future directions

This cross-sectional study was conducted on Iranian patients diagnosed to have βTT and IDA. In this study, two new discriminating indices were proposed for differentiating between the βTT and IDA, and these indices presented a relatively similar diagnostic performance according to cluster analysis compared to different indices reported in the literature. Index26 indicated better performance in comparison with the other discriminating indices. This low-cost index can be useful for differentiating between the βTT and IDA, thus using this index, costs for health system can be minimized in regions with limited financial resources. Also, study results showed that data mining methods like tree-based classification models can be used in order to recommend new discriminating indices for differentiating between the βTT and IDA. CRUISE index was found to have a superior performance compared to some of discriminating indices. This study was also the first study in which cluster analysis was applied for identifying homogeneous subgroups of discriminating indices with similar diagnostic function. Accordingly, it is recommended to use cluster analysis for determining discriminating indices with similar diagnostic performance for future studies.
